# Otite tropicale

**DOI:** 10.48327/KQZA-2C12

**Published:** 2021-02-18

**Authors:** N.I. Lengane, R. Nacanabo, A. Goueta, S. Maiga, E.E.M. Nao, Y.M.C. Gyebre, M. Ouattara, K. Ouoba

**Affiliations:** 1 Service d’ORL et chirurgie cervico-faciale, CHUR de Ouahigouya, BP 16, Ouahigouya, Burkina Faso; 2 Service de pneumologie, CHUR de Ouahigouya, BP 16, Ouahigouya, Burkina Faso; 3 Service d’ORL et chirurgie cervico-faciale, CHU Yalgado Ouédraogo, 03 BP 7022, Ouagadougou, Burkina Faso

**Keywords:** Tuberculose, Otite moyenne, Otorrhée chronique, Tuberculosis, Otitis media, Chronic otorrhea

## Abstract

Un patient de 68 ans présente une otorrhée chronique droite associée à une hypoacousie et une toux chronique. L’otoscopie mettait en évidence des perforations multiples du tympan droit. La recherche de bacilles acido-alcoolo-résistants était positive à l’examen direct des crachats et négative à l’examen du pus d’oreille. Un traitement antituberculeux par voie générale a été initié.

## Abstract (en)

A 68-year-old patient presents with chronic right-sided otorrhea associated with hypoacousis and chronic cough. Otoscopy showed multiple perforations of the right eardrum. The search of acid-fast bacilli was positive for direct examination of sputum and negative for the examination of ear pus. A antituberculosis treatment has been initiated.

## Observation

**Figure 1:**
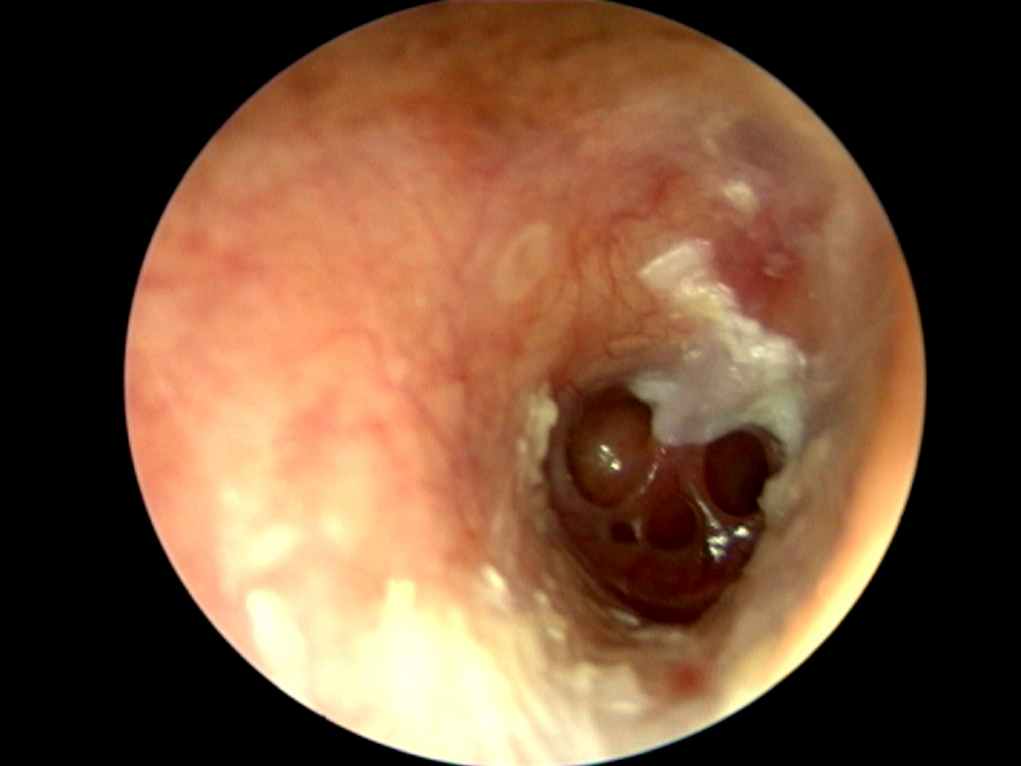
Perforations multiples du tympan droit Multiple perforations of the right eardrum

Un patient de 68 ans est admis au service ORL pour une otorrhée chronique droite associée à une hypoacousie. Le patient était suivi en pneumologie pour une toux chronique. L’otorrhée évoluait depuis 8 mois malgré de nombreuses médications à base d’antibiotiques locaux et par voie générale. Elle était abondante, inodore. L’otoscopie après aspiration et nettoyage du méat acoustique externe mettait en évidence des perforations multiples du tympan droit (Fig. 1). Le tympan gauche était normal.

## RÉponse: Otite moyenne tuberculeuse

La recherche de bacilles acido-alcoolo-résistants était positive à l’examen direct des crachats et négative à l’examen du pus d’oreille. La sérologie rétrovirale était négative. Le patient a bénéficié d’un traitement antituberculeux par voie générale, à base d’isoniazide, de rifampicine, de pyrazinamide, d’éthambutol pendant 2 mois; suivi de l’isoniazide et de la rifampicine pendant 4 mois. L’examen direct des crachats était négatif à 2 mois. L’évolution est marquée par un amendement de l’otorrhée à 1 mois de traitement. L’évolution rapidement favorable de l’otorrhée sous antituberculeux et l’aspect otoscopique nous permettait d’affirmer le diagnostic d’otite moyenne tuberculeuse.

## Discussion

L’otite moyenne tuberculeuse est rare de nos jours. Sa prévalence varie de 0,05 à 0,9 % de l’ensemble des otites moyennes chroniques. Dans certaines communautés la tuberculose peut être responsable de 4 % des otites moyennes. Elle représentait 25 à 50 % des otites moyennes chroniques de l’enfant en 1915. La contamination de l’oreille moyenne se fait directement des poumons, du larynx, du pharynx et du nez à travers la trompe auditive, par voie hématologique, ou par inoculation directe à travers une perforation tympanique [1,6]. L’association à une tuberculose pulmonaire active ou inactive varie de 14 % à 93 %. L’otite moyenne tuberculeuse doit être fortement suspectée chez les patients tuberculeux présentant une infection chronique de l’oreille. L’atteinte est le plus souvent unilatérale [2,7].

La symptomatologie de l’otite moyenne tuberculeuse est semblable à celle des otites chroniques causées par les autres germes. Il s’agit d’une otorrhée chronique qui ne répond pas aux traitements antibiotiques habituels [1]. Les éléments cliniques qui renforcent la suspicion diagnostic est la présence de multiples perforations tympaniques, d’une paralysie faciale, d’une tuberculose pulmonaire, la présence d’un tissu de granulation dans la caisse du tympan. Ces multiples perforations tympaniques correspondent au « tympan en pomme d’arrosoir », hautement évocateur de l’étiologie tuberculeuse. Dans la majeure partie des cas, l’otoscopie met en évidence une vaste perforation centrale. Elle correspond à la convergence de multiples perforations marginales observées autrefois à un stade précoce. Il peut exister des adénopathies periauriculaires [2,3,8,9]. L’exploration radiologique par tomodensitométrie peut mettre en évidence une lyse des osselets et une sclérose de la corticale mastoidienne [2].

Le diagnostic est basé sur l’isolement du germe au microscope ou par culture de prélèvement de l’oreille [1]. La preuve bactériologique est rarement obtenue au niveau auriculaire. L’examen des secrétions auriculaires est positive dans 5 à 35 % des cas [3,8]. Le test rapide de type GeneXpert est une technique rapide et accessible recommandée pour le diagnostic [4]. L’analyse histologique du tissu de granulation prélevé au cours de l’exploration chirurgicale de l’oreille moyenne met en évidence une nécrose caséeuse, des cellules épithéloïdes et des cellules géantes de Langherans [7, 8, 10]. Kameswaran a proposé des critères diagnostiques de l’otite moyenne tuberculeuse. Les critères majeurs comportent une preuve histopathologique, la présence de bacilles acido-alcoolo-résistants au frottis et à la culture du pus d’oreille et un résultat positif à la PCR (polymerase chain reaction). Les critères mineurs sont constitués par la perforation tympanique multiple, le tissu de granulations abondant, la perte auditive sévère, le séquestre osseux, les signes généraux et la paralysie faciale.

Le diagnostic repose sur l’association d’un critère majeur et de deux critères mineurs. En absence de critères majeurs, l’association d’au moins trois critères mineurs indique une forte suspicion diagnostique [10].

Les complications des otites moyennes chroniques tuberculeuses sont rares. Elles peuvent être à type d’abcès cérébraux, de surdité, de paralysie faciale, de labyrinthite, de méningite [1].

Le traitement repose sur un traitement antituberculeux. La chirurgie permet de réaliser un drainage des abcès, et de prendre en charge les complications [1,2,7].

## Conclusion

La tuberculose reste une étiologie exceptionnelle d’otorrhée chronique. Mais il convient de l’évoquer dans notre contexte devant toute symptomatologie trainante résistante aux traitements habituels.

## Conflits D'intérêts

Les auteurs ne déclarent aucun conflit d’intérêts.
